# Local Design Principles at Hippocampal Synapses Revealed by an Energy-Information Trade-Off

**DOI:** 10.1523/ENEURO.0521-19.2020

**Published:** 2020-09-08

**Authors:** Gaurang Mahajan, Suhita Nadkarni

**Affiliations:** Indian Institute of Science Education and Research, Pune 411 008, India

**Keywords:** efficient signaling, hippocampal representation, information theory, short-term plasticity, synaptic facilitation, synaptic failures

## Abstract

Synapses across different brain regions display distinct structure-function relationships. We investigated the interplay of fundamental design constraints that shape the transmission properties of the excitatory CA3-CA1 pyramidal cell connection, a prototypic synapse for studying the mechanisms of learning in the mammalian hippocampus. This small synapse is characterized by probabilistic release of transmitter, which is markedly facilitated in response to naturally occurring trains of action potentials. Based on a physiologically motivated computational model of the rat CA3 presynaptic terminal, we show how unreliability and short-term dynamics of vesicular release work together to regulate the trade-off of information transfer versus energy use. We propose that individual CA3-CA1 synapses are designed to operate near the maximum possible capacity of information transmission in an efficient manner. Experimental measurements reveal a wide range of vesicular release probabilities at hippocampal synapses, which may be a necessary consequence of long-term plasticity and homeostatic mechanisms that manifest as presynaptic modifications of the release probability. We show that the timescales and magnitude of short-term plasticity (STP) render synaptic information transfer nearly independent of differences in release probability. Thus, individual synapses transmit optimally while maintaining a heterogeneous distribution of presynaptic strengths indicative of synaptically-encoded memory representations. Our results support the view that organizing principles that are evident on higher scales of neural organization percolate down to the design of an individual synapse.

## Significance Statement

Synapses across the brain widely vary in morphology and dynamics, suggesting diversity in underlying design principles. The Schaffer collateral-CA1 synapse is a crucial component of the hippocampal circuit associated with learning. We used information transmission and energy utilization, fundamental constraints that govern neural organization, to gain insights into the form-function relationship at this synapse which is characterized by unreliable neurotransmitter release. We show that short-lasting activity-dependent enhancement, a distinguishing attribute of this synapse, ensures that information carried by transmitter release is maximized in an energetically cost-effective manner. Remarkably, we find that synapse-specific quirks ensure information rate is independent of the release probability; thus, even as ongoing long-term memory storage continues to feed heterogeneity in presynaptic strengths, individual synapses maintain robust information transmission. Our analysis reveals the unique design compromises implicit in the distinctive features of this synapse, sharing design principles with higher levels of brain organization.

## Introduction

Chemical synaptic transmission accounts for a significant proportion of metabolic costs during normal neural activity in the mammalian brain (Attwell and Laughlin, 2001). Understanding the role of competing demands imposed by energy consumption and information processing in shaping nervous systems has been an enduring question in neuroscience research (Laughlin, 2001; Balasubramanian et al., 2001; Laughlin and Sejnowski, 2003; Hasenstaub et al., 2010); one may then ask if the notion of energetic efficiency trickles down to the level of individual synapses. In this context, failures of transmitter release, while unsuccessful in relaying presynaptic action potentials, may help conserve synaptic resources by lowering average release rates. Indeed, probabilistic release is a characterizing feature found across a number of synapses (Borst, 2010), and a fundamental source of stochasticity in neural dynamics (Deco et al., 2009). Previous studies have suggested that synaptic failures support both efficient neural coding (Levy and Baxter, 2002) and communication between neurons (Harris et al., 2012), but these studies did not include the effect of use-dependent short-term plasticity (STP) that typically accompanies probabilistic release and which can significantly modulate the time course of synaptic responses to natural activity patterns (Tsodyks and Markram, 1997; Matveev and Wang, 2000; Zucker and Regehr, 2002).

Excitatory Schaffer collateral-CA1 pyramidal cell connections, a crucial component of the hippocampal circuitry engaged during spatial navigation and implicated in experience-dependent learning (Gruart et al., 2006; Basu and Siegelbaum, 2015; Choi et al., 2018), provide a distinctive example of low release probability synapses (Allen and Stevens, 1994). Individual synapses show strong enhancement of release probabilities in response to natural spike trains (Dobrunz and Stevens, 1999), in contrast to their low transmission rates for single spikes; this short-term facilitation (STF) is observed over timescales of milliseconds to seconds. It was previously proposed that the CA3-CA1 pathway is optimized for conveying information on spike times in short bursts occurring at physiologically relevant frequencies (Rotman et al., 2011). However, the concomitant energy costs associated with vesicular release and recycling supporting this form of transmission are not known.

Here, we use a computational model to investigate the relevance of energetic constraints to the design and function of single hippocampal synapses that are characterized by low initial release probabilities but marked activity-dependent STP. Our study addressing individual synapses is thus distinct from earlier work based on synaptic population readouts (Klyachko and Stevens, 2006; Rotman et al., 2011). Other previous studies of information transmission at cortical synapses considered short-time dynamics arising from vesicle depletion alone (Manwani and Koch, 2001; Goldman, 2004; Rosenbaum et al., 2012; Salmasi et al., 2019a), or made simplifying model assumptions about presynaptic organization (e.g., availability of at most one vesicle per release site; Harris et al., 2012; Zhang and Peskin, 2015; Salmasi et al., 2019b), limiting their physiological relevance for describing facilitating hippocampal synapses. Another distinguishing feature of our study is that we do not ascribe a notion of information to “a” spike as is often done (Goldman, 2004; Rotman et al., 2011; Harris et al., 2012), as it is unclear whether every presynaptic spike can be assigned equivalent meaning at the CA3-CA1 synapse. In the hippocampus, neural information may be encoded by brief increases in firing rate rather than the precise timing of individual spikes. A relevant example is provided by the selective activation of specific subsets of CA3 pyramidal cells whenever the animal enters their preferred spatial location (O'Keefe and Dostrovsky, 1971); such brief increases in firing frequency punctuating a low-activity background are regarded as discrete units of information (Lisman, 1997), which may be further modulated by additional task-relevant variables (Fenton and Muller, 1998). Thus, instead of a spike-centric approach assessing how reliably presynaptic spike times are conveyed to the target cell, we make the conceptual distinction that information is more correctly an attribute of the underlying physiologically identifiable temporal “signal” encoded in the irregular firing activity of the presynaptic neuron.

Vesicular release properties are seen to vary widely across synapses, being tuned to the functional demands of the circuits in which they are embedded (Thomson, 2000; Dittman et al., 2000; Abbott and Regehr, 2004). Thus, addressing design principles on a generic level is hindered by the diversity of synapse types found in the nervous system and the neural activity patterns that they process. How a small hippocampal synapse defined by low release probability and a limited pool of available vesicles, equipped with STP, regulates the local balance between reliability and economy of signaling in a physiological setting has not been addressed thus far. Our model includes relevant biological details and characterizes the role of activity-dependent, short-term release dynamics in modulating the transmission of rate-coded behaviorally relevant presynaptic signals at single synapses, providing an apt description of the Schaffer collateral pathway that is characterized by a preponderance of monosynaptic connections between CA3/CA1 cell pairs (Debanne et al., 1995). CA3 synaptic populations display considerable heterogeneity in their transmitter release properties (Allen and Stevens, 1994; Dobrunz and Stevens, 1997; Holderith et al., 2012), and we particularly sought to address how these differences among synapses impact their ability to relay information-carrying spike trains. We show that the vesicle code operates within a range that maintains low energy costs while maximizing information dynamically via STP for the entire heterogeneous population of intrinsic release probabilities seen at these synapses.

## Materials and Methods

**Figure 1.**
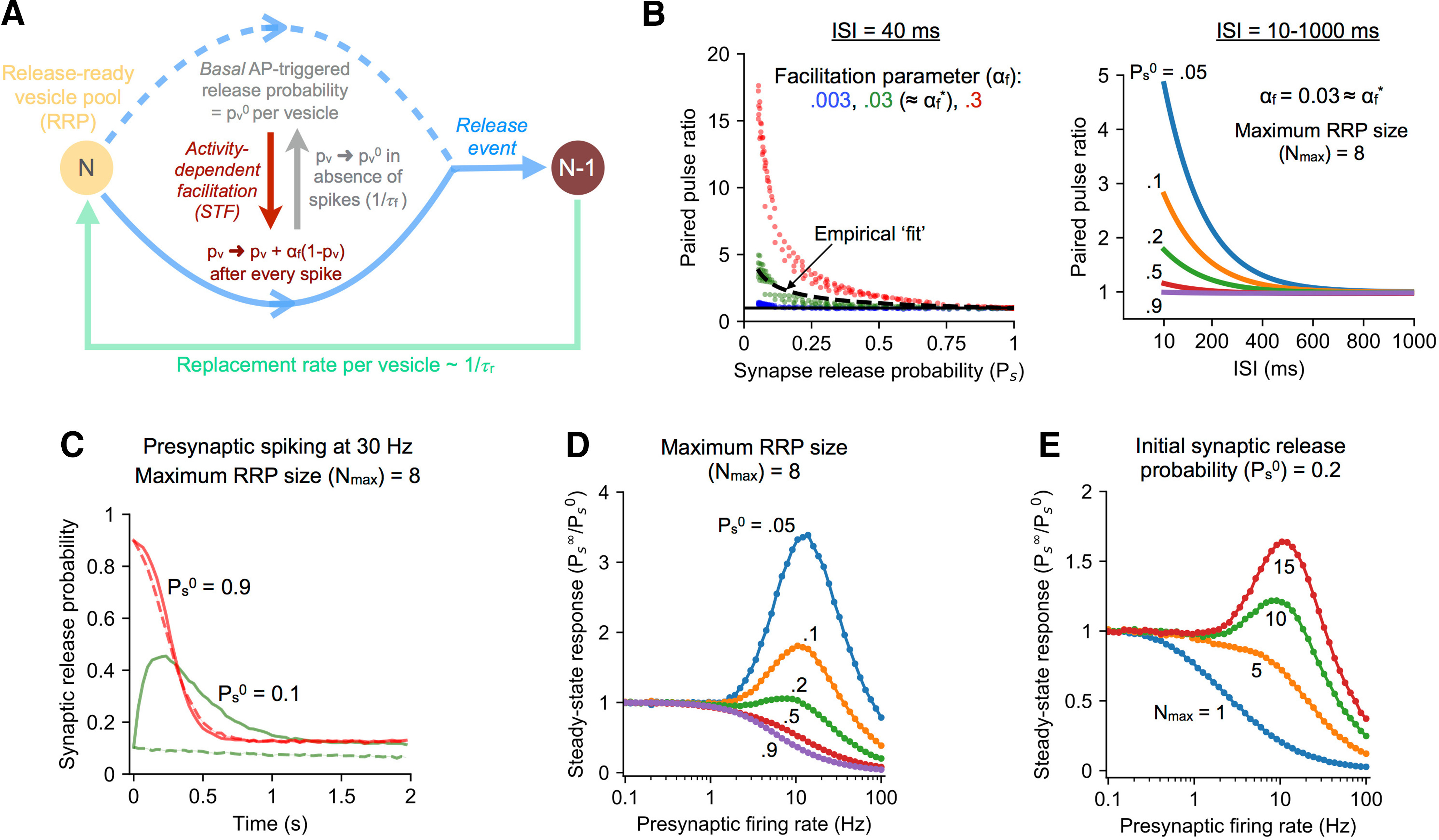
Modeling short-term plasticity (STP) at stochastic hippocampal synapses. ***A***, Outline of the reduced model of presynaptic STP used in the study, which includes activity-dependent facilitation of neurotransmitter release and depression because of slower recovery of released vesicles. Facilitation strength is tuned by the dimensionless gain parameter *α_f_*. ***B***, left, Distribution of PPRs over a realistic range of RRP sizes (1–15) and basal per-vesicle release probabilities ($p_{v}^{0}$ ranging from 10^–4^ to 1) in the STP model (ISI = 40 ms). Different magnitudes of facilitation (*α_f_*) are represented by different colors. The dashed curve captures the empirical distribution of PPR values (from Dobrunz and Stevens, 1997), and the solid black line corresponds to PPR = 1. Right, Dependence of PPR on the interspike interval (ISI) for representative facilitating synapses with the same maximum RRP size and different basal transmitter release probabilities. ***C***, Example of STP dynamics at realistic synapses with low ($P_{s}^{0}$= 0.1) and high ($P_{s}^{0}$= 0.9) initial release probability during the response to sustained presynaptic spiking at 30 Hz. Solid and dashed curves correspond to synapses with facilitation and lacking facilitation (constant *p_v_*), respectively. Results shown as mean ± SEM over 10^4^ independent trials; $N_{\text{max}}$= 8. ***D***, Regimes of facilitation and depression illustrated by the frequency dependence of normalized asymptotic/steady-state response for synapses with different basal failure rates (different colors). All synapses have the same maximum RRP size of 8. Results shown as mean ± SEM over 10^4^ trials per parameter combination. ***E***, Dependence of synaptic filtering on the number of available vesicles illustrated by the frequency-response curves for synapses with the same initial release probability ($P_{s}^{0}$= 0.2) and varying maximum RRP size (different colors represent different $N_{\text{max}}$). Results shown as mean ± SEM over 10^4^ trials per parameter combination.

Experimental studies have provided valuable information about the ultrastructural organization and distribution of dynamic properties at rat CA3-CA1 presynaptic terminals (Dobrunz and Stevens, 1997; Schikorski and Stevens, 1997). Individual CA3 presynaptic boutons typically have a single active zone (Schikorski and Stevens, 1997), where glutamate release occurs in a probabilistic manner (Allen and Stevens, 1994) and shows a complex mix of use-dependent depression because of refractoriness in vesicle recovery and rapid calcium-mediated facilitation of the release machinery (Stevens and Wang, 1995; Dittman et al., 2000). We adopted a mathematical description of this synapse which captures key attributes of its short-time release dynamics (Fig. 1*A*), and quantified through numerical simulations its responses to irregular spike trains mimicking naturally occurring presynaptic cell activity. The model details and setup for our analysis are briefly described below.

### Estimating synaptic information rates

Experimental recordings from rodent hippocampus suggest that individual pyramidal cells in area CA3 show location-specific (place cell) firing during free exploration (Fenton and Muller, 1998; Mizuseki et al., 2012). Further, it has been argued that variability seen in these brief increases in firing during individual passes through the preferred location may encode additional attributes, such as aspects of the animal’s trajectory (Allen et al., 2012; Grieves et al., 2016), its motion relative to goal direction (Aoki et al., 2019), variable attentional state of the animal (Olypher et al., 2002; Fenton et al., 2010), modulation by contextual cues (Shapiro et al., 1997; Leutgeb et al., 2005) (besides simply the variable duration, or equivalently, the running speed, at each pass), etc. These observations motivate our model wherein we consider synaptic processing of integrated spatial and contextual signals represented by the occurrences and variable size (number or frequency of spikes) of place field (PF) discharges of the CA3 neuron. We thus define an effective “input” signal, *ϕ*(t), underlying the variable firing activity of the presynaptic cell which is assumed to be zero everywhere except whenever the trajectory crosses the cell’s preferred location. Every pass through the PF is associated with a non-zero value of *ϕ*, *ϕ_i_*, which in our model is sampled from a uniform random distribution on the interval [$\phi_{\text{min}},\;\phi_{\text{max}}$]. This range is adjusted to be compatible with the statistics of experimentally recorded hippocampal spike train data (described below).

Individual PF passes are assumed to be uncorrelated in time and to occur sparsely, at a mean rate of *r_s_* s^−1^ (hereafter, we use the term “input rate” to refer to the mean rate of PF passes). In order to estimate the information rate carried by *ϕ*(t), time is uniformly divided into sufficiently short steps of Δt (≪$1/r_{s}$), which is taken to be the (fixed) duration of every pass; every time step is thus treated as an independent realization of the probability distribution of *ϕ*, P(*ϕ*), that is determined by the value of *r_s_*. The time-averaged, discretized, entropy rate of the input signal is quantified in the usual manner using Shannon’s measure (Cover and Thomas, 2012):

(1) $$R_{s}=-(1/\Delta t)\sum_{\phi }P(\phi ){\text{log}}_{2}P(\phi )(\text{bits/s}),$$

where the sum runs over all values that *ϕ* can assume, with P(*ϕ*) being approximated by a discrete distribution over n*_ϕ_* possible states (set to 20 in our analysis). The value of *ϕ* during every PF pass determines the corresponding burst size; the number of spikes comprising every burst is thus given by a second, conditional, Poisson distribution with mean *λ*(*ϕ*) (the exact form of which depends on the specific interpretation of the *ϕ* variable; see “Model implementation” below), and these spikes are assumed to occur at random times within the corresponding pass of duration Δt. To be consistent with experimental data, a small amount of “noise” is also added to the system, modeled as a constant background presynaptic spiking rate of *r_n_ s*^−1^ (this spiking is uncorrelated with the spatial context and could arise, say, from synaptic or channel noise).

The synaptic response to presynaptic spike patterns consists of a sequence of evoked transmitter release events, and we quantify how well this discrete temporal sequence conveys the temporal modulation of the signal *ϕ* underlying the irregular firing behavior of the presynaptic neuron. By analogy with the input, we discretize the CA3 synaptic output by binning the releases occurring within every time step Δt. Thus, every burst evokes a variable amount of transmitter, proportional to the number of release events, *n_r_*, and the vesicular response profile is given by a sequence of *n_r_* values (one number per Δt step, and that are assumed to produce graded postsynaptic responses via temporal summation of EPSPs or NMDA receptor-gated Ca^2+^ transients). Under the assumption of low noise level, synaptic transmission is characterized by the stationary joint probability distribution P(*ϕ*, *n_r_*) ≡ P(*ϕ*)P($n_{r}|\phi$) which is (implicitly) sampled at every time step in our simulations. The conditional distribution P($n_{r}|\phi$) is governed by the form of the synaptic dynamics used in the model and encapsulates the effects of STP. Synaptic responses to successive bursts can be considered to be uncorrelated, which is a valid approximation when the typical interval between PF passes is longer than the slowest timescale in the model of synaptic dynamics (this is set by the recovery rate of the release-ready vesicle pool; see below). We characterize the fidelity of information coding at an individual synapse in terms of a discretized version of the average mutual information rate ($R_{rs}$), a standard nonparametric measure of statistical relatedness of two variables, which can be expressed as a difference between the total entropy of the synaptic response and the noise entropy (Cover and Thomas, 2012):

(2) $$R_{rs}=-(1/\Delta t)\times \left[\sum_{n_{r}}P(n_{r}){\text{log}}_{2}P(n_{r})-\sum_{\phi }\sum_{n_{r}}P(\phi )P(n_{r}|\phi ){\text{log}}_{2}P(n_{r}|\phi )\right](\text{bits/s}).$$

This rate is numerically estimated from the pooled data from simulations run for sufficiently long duration.

### Estimating efficiency of the vesicle code

Following previous studies (Goldman, 2004; Harris et al., 2012; James et al., 2019), local energetic efficiency of the synaptic code is quantified in terms of the average number of releases per bit transmitted per synapse (i.e., the specific cost of information), and we use the measure $E(s^{-1})=R_{ves}/(R_{rs}/R_{s})$, where $R_{ves}$ denotes the mean rate of fusion events at the synapse (averaged over every simulation run). $R_{ves}$ accounts for the use of synaptic resources during signal transmission and also provides a proxy for the net energy expenditure associated with transmitter release and recycling (Harris et al., 2012). The specific or unit cost of information as defined above is inversely related to the notion of efficiency, i.e., higher efficiency synapses are expected to require fewer vesicles to carry information at a given rate.

### Modeling release dynamics at probabilistic synapses

Every synaptic release site is characterized by its basal spike-evoked transmission probability, $P_{s}^{0}$, and maximum size of the docked pool of release-ready vesicles [readily-releasable pool (RRP)], $N_{\text{max}}$. The synaptic release probability (*P_s_*) is distinct from the fusion probability per docked vesicle (*p_v_*), and under the assumption that docked vesicles can fuse independently of each other (Dobrunz and Stevens, 1997), the two are related by *P_s_* = 1 – (1 – *p_v_*)${}^{N_{\text{RRP}}},\;N_{\text{RRP}}$ being the instantaneous RRP size at the release site.

We model the synchronous component of vesicular release evoked by presynaptic spikes (Dobrunz and Stevens, 1997) and assume that every spike can trigger release of at most one vesicle per synapse. This assumption of uniquantal release at glutamatergic CA3-CA1 synapses is compatible with experimental findings (Stevens and Wang, 1995) suggesting a refractory phase associated with fusion of a vesicle, which may inhibit subsequent release events in a short time period (∼10 ms) following the initial spike during which the local calcium concentration at the release site is high. Following its release, every vesicle is assumed to be recovered independently, and this replenishment is also modeled as a stochastic process with mean recovery timescale of *τ_r_* per vesicle. During bouts of intense spiking activity, rapid depletion of the docked vesicle pool can occur, mediating a form of transient depression whose strength and duration are controlled by *τ_r_* together with the resting pool size at the synapse. We set *τ_r_* to 2 s, consistent with experimentally measured refilling rates at rat hippocampal synapses (Klyachko and Stevens, 2006).

Transient depression because of slow recycling of released vesicles is complemented by activity-dependent changes in vesicle fusion at the release site. We adopt a reduced kinetic model (Fig. 1*A*) to describe this STF regulated by spike-driven calcium dynamics at the active zone, which follows from a number of previous studies of presynaptic plasticity (Tsodyks and Markram, 1997; Hennig, 2013). The spike-triggered per-vesicle release probability, *p_v_*, is treated as a dynamical variable whose dependence on the presynaptic spiking history is governed by the following equation:

(3) $$\frac{dp_{v}}{dt}=\frac{\left(p_{v}^{0}-p_{v}\right)}{\tau_{f}}+\alpha_{f}(1-p_{v})\sum_{i}\delta (t-t_{i}),$$

with the sum running over the set of all spike times. Thus, the arrival of every presynaptic spike increments the value of *p_v_* by an amount proportional to the synaptic gain parameter *α_f_*, and the factor (1 – *p_v_*) ensures that *p_v_*, being a probability, does not exceed 1. The first term on the RHS of Equation 1 describes the exponential relaxation of *p_v_* to its baseline value $p_{v}^{0}$ in the absence of spiking activity. Interspike intervals shorter than the facilitation time constant, *τ_f_*, are expected to induce strong enhancement of vesicle release. Following previous studies, we model a rapid form of STF with *τ_f_* = 150 ms (Klyachko and Stevens, 2006; Cai et al., 2007). Longer-lasting forms of presynaptic plasticity such as augmentation are not considered here; these components are normally induced in experimental settings with sustained high-frequency synaptic stimulation (Zucker and Regehr, 2002), and unlikely to be of significance during the sparse, sporadic spiking activity observed in the physiological conditions modeled here.

The form of STF given by Equation 3 implies a general trend of decreasing facilitation with increase in the basal synaptic release efficacy, which is supported by experimental recordings of individual hippocampal synapses (Dobrunz and Stevens, 1997; Murthy et al., 1997). In order to select a physiologically relevant value for the gain parameter *α_f_*, we refer to previous experimental data on paired-pulse stimulation at rat CA3-CA1 synapses [Dobrunz and Stevens, 1997; sex of animal(s) not mentioned in source publication]. This synaptic population displayed a broad range of baseline *P_s_* values (∼0.05–1), and the release probabilities recorded in response to two spikes separated by a short interval (40 ms) yielded a distribution of paired-pulse facilitation ratios (PPRs) whose dependence on the initial synaptic release probability (*P_s_*) was well-fit by the relation PPR = (1 – (1 – *P_s_*) ${}^{aP_{s}^{b}}$)/*P_s_* (a = 1.24 ± 0.15, b = −0.41 ± 0.05). Using our simplified description of presynaptic dynamics, we analytically estimated the PPR in our model over a realistic range of basal per-vesicle release probabilities ($p_{v}^{0}$ = 10^– 4^ to 1) and RRP sizes ($N_{\text{max}}$ = 1–15), and found the value of *α_f_* for which this distribution of values was optimally fit by the above empirical model. The mean-squared error was minimized at *α_f_* ≈ 0.03. We thus set $\alpha_{f}^{*}$ = 0.03 as the biological reference value of the gain parameter in our simulations, and, separately, also examined the effects of reducing or increasing the level of facilitation on synaptic coding properties (Fig. 1*B*).

### Model implementation

Realistic values of various parameters for the input stimulus were chosen in accordance with *in vivo* CA3 spike recordings from awake, freely moving rodents (adult male Long–Evans rats; Fenton and Muller, 1998), which have been used in previous STP studies (Klyachko and Stevens, 2006; Kandaswamy et al., 2010; Rotman et al., 2011). This dataset comprises inhomogeneous spike trains spanning a broad range of discharge frequencies (∼5–60 Hz) and burst sizes (∼3–30 spikes per burst), with typically long (approximately several seconds) quiescent periods separating individual discharges. We modeled two specific implementations of the temporal signal *ϕ*(t) shaping presynaptic spiking activity: one describing frequency modulation of sporadic PF firing (rate remapping), and another wherein it represents the variable duration (with fixed discharge frequency) of individual PF passes. Non-zero instances of *ϕ* were sampled randomly from an appropriate dynamic range accordingly. For the variable frequency case, spike rate for every pass was sampled from the 6- to 60-Hz range, and the step size was set to Δ*t* = 0.5 s, which also gives a consistent range of spike numbers per burst (3–30 on average). Alternately, variable-duration passes were modeled with a fixed in-field firing rate of 30 Hz (the average discharge frequency from experimental recordings) and individual passes spanning 0.1–1 s, again giving between 3 and 30 spikes per burst on average. Further, to simplify estimation of information rates in this case, the step size was fixed at Δ*t* = 0.1 s, and every time a PF pass was reckoned to occur, that step was assigned a variable number of spikes, based on the duration (value of *ϕ*) corresponding to that instance. Thus, variable duration bursts were rescaled to a constant step Δt; this is a valid approximation when estimating average information rates over very long times, provided that burst durations ≪1/*r_s_*, which is compatible with the available data. To reduce errors in estimation of $R_{\text{info}}$ in this approximation, facilitation and refilling time constants were also appropriately rescaled when implementing STP dynamics within every PF crossing by the corresponding duration *t_B_* (*τ_f_* → $\tau_{f}\Delta t$/*t_B_* and *τ_r_* → $\tau_{r}\Delta t$/*t_B_*), to mitigate over- (under-)estimating the effect of activity-dependent facilitation (vesicle recycling).

Monte Carlo simulations of the STP model were conducted for a range of input rates (*r_s_* = 0.05–0.2 *s*^−1^), low noise levels (*r_n_* = 0–1 *s*^−1^) and maximum RRP sizes ($N_{\text{max}}$ = 1–15), and dependence of results on the basal $p_{v}^{0}$ was characterized over approximately four orders of magnitude ($p_{v}^{0}$ = 0.0001–1). For every distinct combination of parameter values, 20 independent runs (each of 3 × 10^4^ s in duration) were simulated, and the time-averaged rates of information flow and release events were estimated for every trial. The averages from our simulations were found to provide accurate estimates of the asymptotic information rates, justifying comparisons between different synaptic configurations in terms of the corresponding across-trial averages. To specifically assess the differential effect of STF on synaptic function, every synapse with STF (referred to as a dynamic synapse) was compared with an equivalent static synapse which lacks facilitation while still exhibiting activity-dependent vesicle depletion (this corresponds to setting *α_f_* = 0 in Eq. 3). In the following, only the results for the model with variable burst frequency are presented, although we have separately verified that the findings are closely reproduced for the variable-duration model as well.

#### Code accessibility

All simulations, data analysis, and visualization were performed in Python using the NumPy, SciPy, and Matplotlib modules. Simulations were carried out on a desktop PC (Intel Xeon CPU @ 3.5 GHz × 8, 32 GB RAM) running Ubuntu 16.04 operating system. Basic code implementing the model is freely available online at GitHub (https://github.com/gmcoderepo/stp_at_ca3synapse). The code is available as a Python file in Extended Data 1.

10.1523/ENEURO.0521-19.2020.ed1 Extended Data 1 Python 2.7 code implementing the STP model (stpmodel.py). Download Extended Data 1, ZIP file.

## Results

### Improved signaling at unreliable synapses with STF

How does STF shape the vesicle code conveying information about presynaptic cell activity at stochastic hippocampal synapses? Experimental measurements reveal considerable diversity in the RRP size, release probability and STP properties across individual CA3-CA1 synapses (Dobrunz and Stevens, 1997; Schikorski and Stevens, 1997) that is invoked in our synaptic model (outlined in Materials and Methods and Fig. 1*B*). To explore the role of various synaptic attributes in modulating its transmission properties, we simulated the synaptic response to regular presynaptic spike trains occurring at different rates. Figure 1*C* shows the response of a synapse to persistent spiking at 30 Hz (the average frequency in experimentally recorded bursts) for low and high initial release rates ($P_{s}^{0}$ = 0.1 and 0.9) and a canonical maximum RRP size of $N_{\text{max}}$ = 8. For the low *P_s_* synapse, the time-dependent release probability initially increases because of strong activity-dependent facilitation and reaches a maximum, before the effect of vesicle depletion and slower recovery takes over, causing a drop in the response which eventually settles at a steady state value determined by the firing frequency. In contrast, the high *P_s_* synapse shows a monotonically decreasing response with time, as it undergoes weaker facilitation and a larger initial *P_s_* also implies faster depletion of the readily-releasable vesicle pool. The above qualitative differences illustrate the regimes of synaptic enhancement and depression encompassed by the STP model (Fig. 1*A*) that is based on physiological parameters for facilitation and depletion. To further bring out the differences between these two limits, we quantified the asymptotic/steady-state response amplitude of the dynamic synapse to input trains spanning a wide range of frequencies (0.1–100 Hz) as a function of the initial transmitter release probability. Figure 1*D* shows the normalized synaptic response profiles for different base synaptic failure rates at a fixed RRP size ($N_{\text{max}}$ = 8). High *P_s_* synapses are most effective at transmitting spikes arriving at low frequencies, and with increasing facilitation (lower *P_s_*), the optimal transmission frequency is shifted to higher frequencies, demonstrating a transition from depression-dominated to facilitation-dominated behavior governed by the overall nature of STP dynamics (Eq. 3).

It is to be noted that RRP size also influences synaptic behavior along with the value of $P_{s}^{0}$, the latter being a function of the maximum number of vesicles available for release as well as the basal per-vesicle fusion probability ($p_{v}^{0}$). To demonstrate the role of RRP size in modulating synaptic behavior, the frequency-response relation estimated for different numbers of release-ready vesicles with a fixed basal synaptic release rate ($P_{s}^{0}$ = 0.2) is shown in Figure 1*E*. These curves highlight the role of RRP size in tuning the response profile of the synapse: synapses with a given fixed initial probability of release ($P_{s}^{0}$) but differing in their number of available vesicles show a range of responses, from low-pass filtering for smaller $N_{\text{max}}$ (corresponding to high basal $p_{v}^{0}$) to higher optimal transmission frequencies for larger RRP sizes (lower basal $p_{v}^{0}$). Taken together, the above examples (Fig. 1*C-E*) capture the broad repertoire of behavior displayed across an ensemble of facilitating probabilistic synapses in the physiological regime (Dobrunz and Stevens, 1997), that is controlled by the interplay among key synaptic parameters governing transmitter release and recovery.

**Figure 2.**
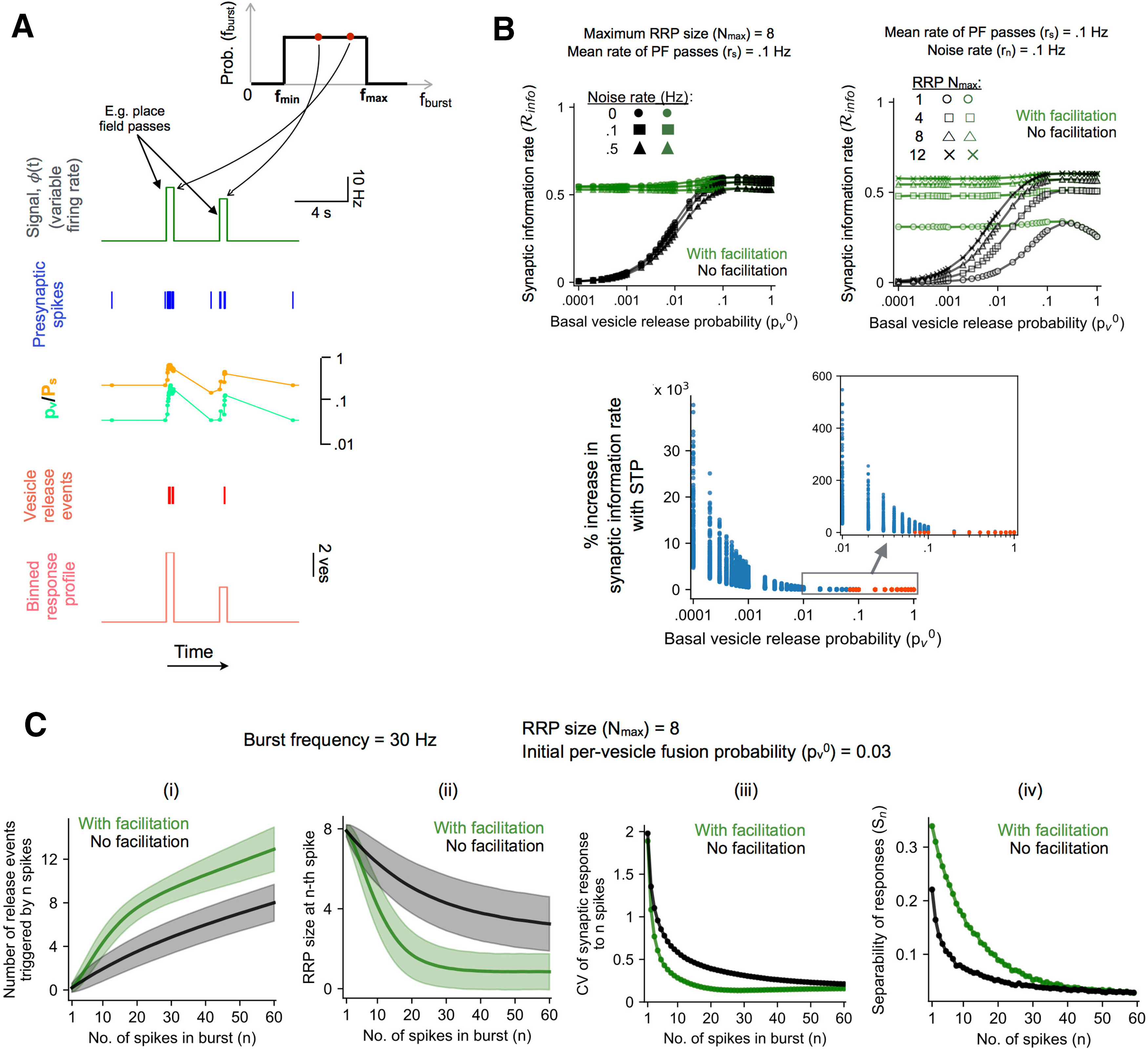
Elevated transmission of presynaptic information with STP of vesicular release. ***A***, Time trace illustrating the conversion of an input signal [*ϕ*(t)] to a sequence of synaptic release events governed by STP. For this example, $P_{s}^{0}$= 0.2, $N_{\text{max}}$ = 8, and *r_s_* = *r_n_* = 0.1 *s*^–1^. The uniform distribution of spiking frequencies from which individual burst instances are randomly sampled is shown in the top right corner. ***B***, top left, Time-averaged rate of information carried by synaptic release events ($R_{\text{info}}$) as a function of the basal per-vesicle fusion probability ($p_{v}^{0}$) for a synapse with STF (green curves) and an equivalent non-facilitating synapse (black curves). Different markers indicate different noise levels. (Results shown as mean ± SEM over 20 independent simulations; $N_{\text{max}}$= 8 and input rate *r_s_* = 0.1 *s*.) Top right, Synaptic information transfer rates with STP (green curves) and without facilitation (black curves) for different numbers of available vesicles (different markers). (Results shown as mean ± SEM over 20 independent trials; *r_n_* = *r_s_* = 0.1 *s*^–1^.) Error bars being smaller than the marker size are not visible. Bottom, Enhancement of synaptic information transmission with facilitation summarized as a distribution of relative changes (% difference of means relative to the static synapse) over a biologically relevant range of input/model parameters (for details, see Materials and Methods). Inset shows a magnified view of the 0.01 $\leq p_{v}^{0}\leq$ 1 interval. Color coding indicates statistical significance of pairwise differences (blue: significant at FDR <0.001 level, red: not significant). ***C***, Synaptic response (i) and progressive depletion of the readily releasable vesicle pool (ii) as functions of the number of input spikes for a synapse with STF (green) and lacking facilitation (black). Response fluctuations are quantified in terms of the CV of number of release events (iii) and separability of responses to bursts differing in size by a single spike (iv). Results in i, ii shown as mean ± SD (1000 independent trials); $P_{s}^{0}$ = 0.2, $N_{\text{max}}$ = 8, and spikes are Poisson-distributed with mean frequency of 30 Hz.

Naturally occurring firing patterns in the CA3 region are characterized by brief increases in firing frequency (spike bursts) separated by long periods of low activity (Klyachko and Stevens, 2006) and were shown to encode behaviorally relevant integrated spatial and contextual signals. These observations inform our model, and we next examine synaptic processing of spike trains mimicking such activity patterns. Figure 2*A* illustrates the steps involved in our simulation of STP dynamics for a synapse with $P_{s}^{0}$ = 0.2 when the presynaptic spiking pattern carries information in the temporal sequence of burst occurrences and the variable firing frequency associated with every burst. The stochastic, time-varying signal (effectively an inhomogeneous Poisson process) is reflected in the brief spike discharges of the presynaptic neuron during passages through its preferred location (PF); this spiking activity drives the temporal dynamics of *p_v_* and the transmitter release probability (*P_s_*), eliciting a sequence of vesicular release events. We used a binning procedure to estimate the mean information content in the quantal release profile about the presynaptic signal (for details, see Materials and Methods). Figure 2*B*, top left, shows the relative mutual information rate, $R_{\text{info}}\;\equiv \;R_{rs}/R_{s}$, as a function of the basal probability of vesicle release ($p_{v}^{0}$) for different noise levels, for both a canonical synapse exhibiting STP (green lines) and an equivalent synapse which does not show facilitation (black lines). Figure 2*B*, top right, illustrates the dependence of synaptic information rates on the RRP size for a fixed noise level of *r_n_* = 0.1 Hz. These examples indicate a general enhancement of synaptic information transfer with STF, significantly expanding the scope of previous results from spike-based studies (Pfister et al., 2010; Rotman et al., 2011). Further, this increase is more pronounced for synapses with lower release probability, aligning with our expectation that smaller basal $p_{v}^{0}$ combined with stronger facilitation (Fig. 1*B*) accentuates the differential response to bursts versus single spikes, enhancing the ability of the synapse to selectively transmit information-carrying brief, high-frequency spike discharges. To address the generality of the above results, we repeated our simulations over a biologically relevant range of parameter values (input rate/mean rate of PF passes = 0.05–0.2 Hz, noise level = 0–1 Hz and RRP size = 1–15). The overall difference in synaptic information capacity in the presence and absence of STF is summarized as a distribution of relative changes in $R_{\text{info}}$ (percent difference of means) in Figure 2*B*, bottom, and the color coding represents statistical significance of pairwise differences [two-sided Wilcoxon rank-sum test followed by Benjamini–Hochberg adjustment for multiple comparisons; blue: significant at false discovery rate (FDR) <0.001, red: not significant]. STF is found to robustly improve the fidelity of synaptic signaling in the physiological regime. The differential effect of facilitation scales inversely with the basal synaptic release efficacy, and is more marked for higher synaptic failure rates (lower $p_{v}^{0}$). The effect of facilitation is diminished with increase in the basal release probability, and there is little difference in transmission efficacy between the dynamic and static synapses for $p_{v}^{0}$ values above ∼0.1.

How can this improved reliability of synaptic information transmission be understood in simple terms? We recall that the synaptic response to information-carrying brief spike discharges consists of a variable number of release events; thus, the information content of the vesicle code in our formulation is essentially determined by how well the different output sizes (total number of releases triggered by a spike discharge) can discriminate between different input states (i.e., the variable spiking frequency, or duration, associated with every burst). We characterize the reliability of this mapping in terms of the cumulative number of released vesicles as a function of the burst size (number of spikes). Figure 2*Ci* compares the responses of a canonical synapse (basal $p_{v}^{0}$ = 0.03 and maximum RRP size = 8) with and without STF to Poisson spiking at a mean rate of 30 Hz. In the presence of facilitation, not only is the average response amplitude (total number of release events for a given burst size) larger, but more importantly, it is also a more reliable readout of the burst size, because of reduced variability of responses relative to the static synapse. This difference is clearly seen in Figure 2*Ciii*,*iv*, where two distinct measures for response fluctuations are plotted as functions of the number of spikes (*n*). The coefficient of variation (CV; defined as the standard deviation of the response relative to its mean) is lower for the synapse with facilitation, and the separability, defined as *S_n_* = ($\mu_{n+1}$ − *μ_n_*)/($\sigma_{n+1}$ + *σ_n_*) (*μ_n_* and *σ_n_* denoting the mean and SD, respectively, of the total response to *n* spikes), is correspondingly larger. The initial steeper increase in the mean response with the number of spikes (Fig. 2*Ci*), together with smaller dispersion of responses (Fig. 2*Ciii*,*iv*), implies better correspondence between the response amplitude and the burst length when STF is included. It is to be noted, though, that this increased reliability is also accompanied by faster depletion of the readily-releasable vesicle pool at the facilitating synapse (Fig. 2*Cii*), implying a reduced dynamic range of burst sizes that can be conveyed by synaptic release events (this is indicated by a sharp change in the slope of the response profile for the facilitating synapse beyond some threshold spike number in Fig. 2*Ci*). However, our results indicate that, in the biologically relevant parameter range considered here, this downside of STP is more than offset by the advantage of increased reliability of responses to the shorter bursts (Fig. 2*Ciii*,*iv*) in the presence of facilitation, leading to significant net improvement in synaptic information transfer (Fig. 2*B*). In sum, our simulations of STP dynamics highlight a crucial functional role for activity-dependent facilitation at unreliable hippocampal synapses, in conferring distinct advantage for the transmission of information represented by time-varying presynaptic cell activity in a physiologically relevant setting.

### Reliable signaling at realistic STP synapses is nearly independent of their basal release properties

The results in the previous section (Fig. 2*B*) indicate that, for realistic number of vesicles, the signaling capacity of an STP synapse is not only increased relative to a static synapse, but, notably, also independent of its initial per-vesicle release rate ($p_{v}^{0}$) for the characteristic inhomogeneous spiking patterns associated with CA3 pyramidal cells studied here. To elaborate on the dependence of synaptic information transfer on its basal transmission probability, we scaled the mean information rate estimated for each value of $p_{v}^{0}$ by the maximum value attained across the full range of $p_{v}^{0}$ values considered (this was done separately for every combination of input rate, noise rate, and vesicle pool size). This rescaling factors out the dependence on the other model parameters, and reveals the general trend in dependence of synaptic information transfer on its intrinsic reliability.

**Figure 3.**
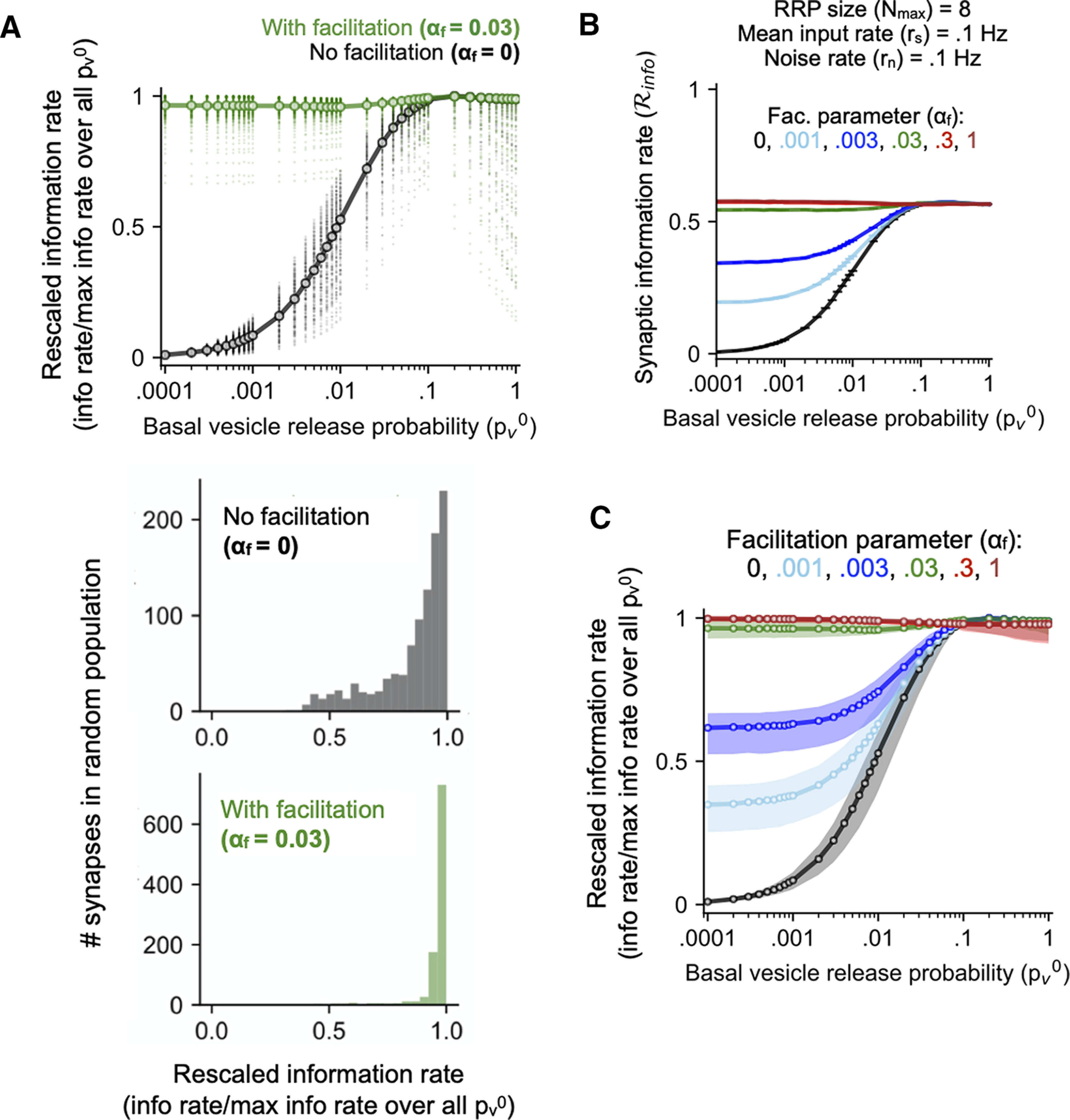
Information transmission properties in an ensemble of facilitating CA3-CA1 synapses. ***A***, top, Dependence of information rate estimates (rescaled values) on the basal probability of vesicle release ($p_{v}^{0}$) for synapses with physiological facilitation strength (green) and non-facilitating synapses (black) over a realistic range of input/model parameters (for details, see Materials and Methods). Every point represents a distinct parameter combination, and continuous lines connect the medians (one per value of $p_{v}^{0}$). Bottom, Distribution of rescaled information rates in a representative population of static (black) and facilitating (green) synapses with variable per-vesicle basal release probability ($p_{v}^{0}$; *n* = 1000 synapses, randomly sampled from 0.05 $\leq P_{s}^{0}\leq 0.6$ and 1 $\leq N_{\text{max}}\leq$ 15; input rate and noise rate were also randomly set for each synapse). ***B***, Estimated time-averaged information transfer rate as a function of the initial per-vesicle release probability ($p_{v}^{0}$) for synapses with different levels of facilitation (approximately three decades in the facilitation parameter *α_f_*). Results shown as mean ± SEM (20 independent trials) for each choice of *α_f_*. $N_{\text{max}}$ = 8; rate of burst occurrences = noise rate = 0.1 *s*^–1^. The non-facilitating static synapse is shown in black. ***C***, Distributions of rescaled information rates over a realistic range of inputs/model parameters for different magnitudes of the synaptic gain *α_f_* (0.001–1). Each distribution is displayed in terms of the medians and 25th –75th percentile (interquartile) ranges (per value of $p_{v}^{0}$).

Figure 3*A*, top, shows the distribution of rescaled information capacity values separately for synapses with STF (green points) and lacking facilitation (gray points); each point represents a particular combination of model parameter values and $p_{v}^{0}$. These results indicate that synaptic information coding is robust to differences in the basal *p_v_* at dynamic synapses. In other words, STP ensures that synapses with widely different basal fusion probabilities transmit at comparable rates (median of values for each $p_{v}^{0}≳$ 95% over the full range of $p_{v}^{0}$ values considered here). By contrast, information carried by synapses lacking facilitation shows clear dependence on the magnitude of the basal *p_v_*, and is strongly impaired for synapses with $p_{v}^{0}\;≲\;$0.1.

These data are especially interesting in the light of the considerable heterogeneity in the magnitude of $p_{v}^{0}$ at hippocampal CA3 synaptic populations as reported by experimental studies (Dobrunz and Stevens, 1997; Murthy et al., 1997; Holderith et al., 2012). Our analysis indicates that synaptic information transfer in the presence of dynamic gain control (Eq. 3) is nearly invariant to differences in the basal fusion probability per vesicle (Fig. 3*A*, bottom), and there is no appreciable change in the overall information transfer profile over nearly four orders of $p_{v}^{0}$ (Fig. 3*A*, top). Recalling that the information rate (Eq. 2) provides a measure of how well the different presynaptic input states (spiking frequencies) are discriminated by the output states (total transmitter release per spike burst), the form of STP (Eq. 3) with physiological facilitation strength is such that low release probability synapses achieve a discriminability comparable to synapses with more reliable release. Although we have demonstrated this for a specific dynamic range of CA3 spiking frequencies consistent with available experimental data, the invariance of synaptic information capacity to the basal release probability is found to hold fairly robustly over a range of input distributions [maximum burst frequency (f*_max_*) ≳ 40 Hz; data not shown]. Thus, we propose that physiologically realistic STP works to counteract degradation of presynaptic signals at synapses with small release probabilities, and enables CA3-CA1 synapses to maintain stable information rates in the face of necessary heterogeneity in the basal *p_v_*, arising from long-term changes associated with learning or homeostatic plasticity mechanisms on the cellular/network level.

The above analysis reveals significant overall difference in the profile of information transfer at stochastic synapses in the presence of STF (Fig. 3*A*). How sensitive are these effects to its magnitude? Recalling that the dynamics of the release probability in our effective description of STP (Eq. 3) is essentially controlled by the gain parameter *α_f_*, which was adjusted to be compatible with experimental findings, we ask how the behavior of synapses changes for weaker or stronger facilitation. Figure 3*B* shows an example of the (unscaled) synaptic information rate (mean ± SEM) as a function of the basal $p_{v}^{0}$ for a synapse with $N_{\text{max}}$ = 8 vesicles, across a range of *α_f_* values spanning approximately three orders of magnitude (0.001–1). Figure 3*C* compares the distributions of the rescaled information rate (estimated as before over a broad range of model parameters) for different facilitation strengths. The dispersion of estimates for each $p_{v}^{0}$ is represented in terms of the median (solid line) ± interquartile range (IQR) separately for every *α_f_*. These results indicate that the profile of scaled synaptic information capacity is strongly modulated by changes in *α_f_* especially at the smaller $p_{v}^{0}$ values (≲0.1). This is a reflection of the greater sensitivity of facilitation at smaller basal release probabilities to changes in *α_f_* in the STF model (Fig. 1*B*). In particular, reduction of *α_f_* below the biological estimate ($\alpha_{f}^{*}$) suppresses information transfer for smaller release probabilities and introduces heterogeneity in the ensemble behavior, whereas for *α_f_* $≳\alpha_{f}^{*}$, the rescaled information capacity is nearly independent of the basal synaptic failure rate.

### Short-term release dynamics regulates the capacity-cost trade-off at probabilistic synapses

**Figure 4.**
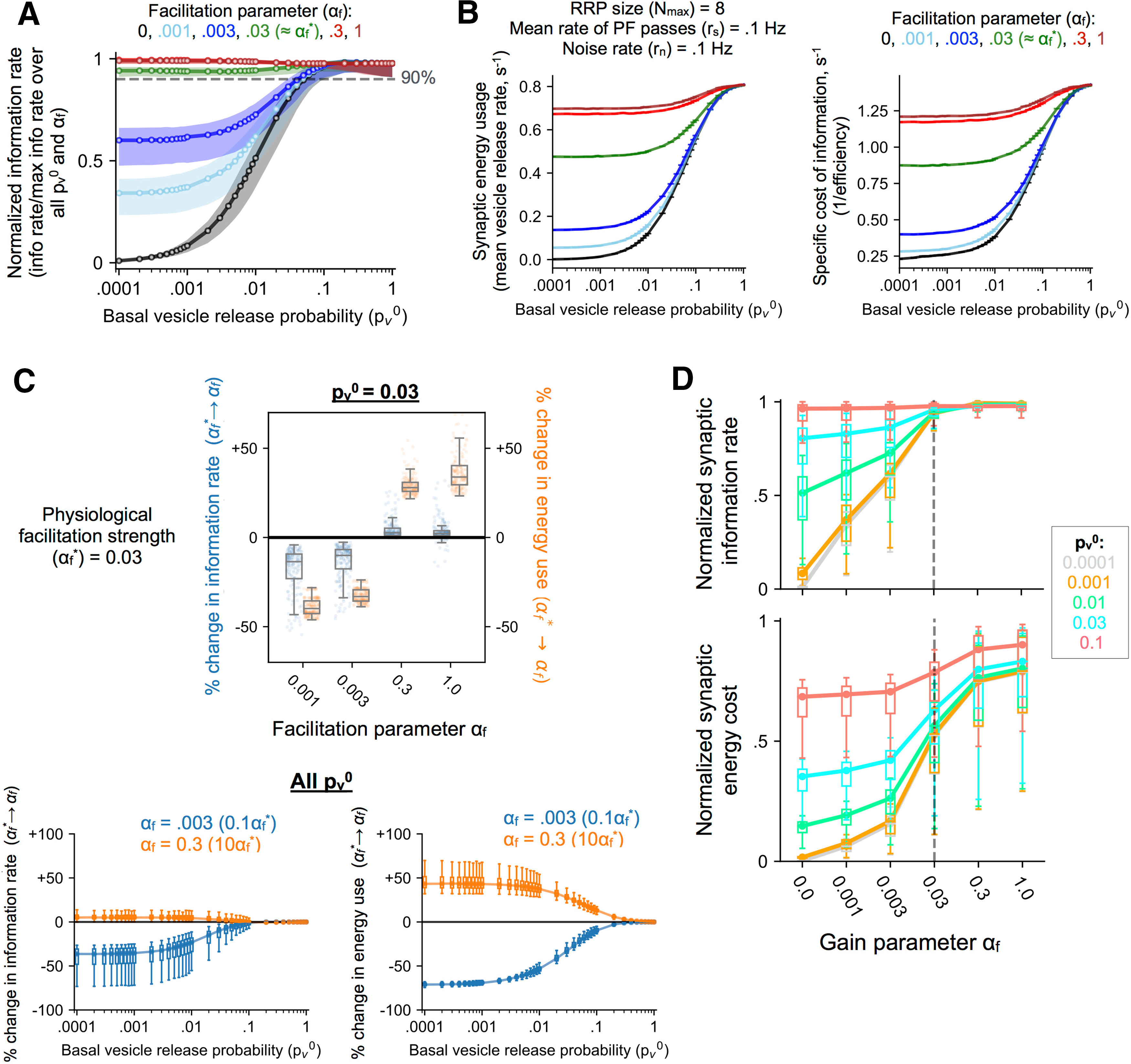
Optimal signaling and synaptic energy efficiency with physiologically realistic STP dynamics. ***A***, Distributions of normalized synaptic information rate (fraction of maximum capacity) over a biologically relevant range of parameters for different choices of the facilitation parameter *α_f_* (different colors). Each distribution is displayed in terms of the data medians and IQRs over a broad range of $p_{v}^{0}$ values. Realistic synapses ($\alpha_{f}^{*}$ ≈ 0.03) transmit at close to maximum capacity overall (median values > 90% across all $p_{v}^{0}$). ***B***, Example profiles of the time-averaged energy usage (left) and the specific cost of information transfer, i.e., inverse of the efficiency (right) for synapses with different facilitation strengths (all other parameter settings are same as in the example in Fig. 3*B*). Data shown as mean ± SEM (20 independent simulations). ***C***, top, Box-plots of relative changes (%) in synaptic information capacity and energy requirement when the synaptic gain is scaled either up or down by a factor of ∼10 relative to its physiological level ($\alpha_{f}^{*}$) for a synapse with basal $p_{v}^{0}$ = 0.03. Each distribution covers a biologically relevant range of input parameters and maximum RRP sizes (*n* = 180 points; for details, see Materials and Methods). Bottom, Summary statistics of relative changes (%) in synaptic information rate (left) and average frequency of release events (right) when *α_f_* is scaled up (orange) or down (blue) by 10×, for a wide range of $p_{v}^{0}$ values. ***D***, Estimates of normalized information capacity (top) and normalized energy expenditure (bottom) as functions of the synaptic facilitation strength *α_f_* which spans approximately three orders of magnitude (also shown, for reference, are results for the static synapse, corresponding to *α_f_* = 0). Each box summarizes the results over a biologically relevant range of input/model parameters, and lines connect the median values for each choice of *α_f_*; profiles for different choices of the basal $p_{v}^{0}$ are represented by different colors. Vertical dashed lines highlight the biological operating point ($\alpha_{f}^{*}\approx$ 0.03).

In the previous section, we showed that STF, in general, enables probabilistic synapses to signal the occurrence and length of brief high-frequency spike discharges more reliably. What is the theoretical limit on synaptic information capacity achievable at individual facilitating synapses, when transmitter release is governed by the STP model analyzed here (Fig. 1*A*)? For every combination of stimulus rate (*r_s_*), noise (*r_n_*), and RRP size ($N_{\text{max}}$), we estimated the maximum rate of synaptic information transfer attainable when *α_f_* and $p_{v}^{0}$ are allowed to vary, and we examined how well biological synapses (corresponding to *α_f_* = $\alpha_{f}^{*}$) compare against this upper bound on $R_{\text{info}}$ (denoted as $R_{\text{info}}^{*}$). Figure 4*A* displays the distributions of the normalized channel capacity ($R_{\text{info}}$/$R_{\text{info}}^{*}$) for different choices of *α_f_* (different colors); for every $p_{v}^{0}$, the distribution over a range of input parameters and RRP sizes is represented in terms of the median and IQR. Our results show that biological synapses (*α_f_* $\approx \alpha_{f}^{*}$) uniformly reach high, near-optimal, information rates under physiological conditions over approximately four orders of magnitude of the basal per-vesicle release probability examined here (the median of normalized estimates for each $p_{v}^{0}$ exceeds 90% over the full range of $p_{v}^{0}$ values considered). By contrast, probabilistic synapses with weaker facilitation (by a factor of 10 relative to the physiological level), or no facilitation altogether, are much less effective at conveying information about presynaptic spiking activity, and the fidelity of information transfer at these synapses is markedly suppressed for $p_{v}^{0}\;≲\;$0.1 (Fig. 4*A*).

Previous studies have emphasized the relevance of energetic constraints for a better understanding of neurobiological design on diverse scales (Laughlin, 2001; Laughlin and Sejnowski, 2003); examples from sensory systems, in particular, suggest that synaptic function may be significantly influenced by energy (resource) limitations (Laughlin et al., 1998; Harris et al., 2015). To evaluate the potential role of energy constraints in shaping synaptic information processing in the hippocampus, we revisit the example in Figure 3*B*, and quantify the synaptic resource use vis-à-vis information transfer at individual facilitating synapses. Figure 4*B* shows the dependence of the average vesicular release rate and the energy efficiency of information transduction (∼average number of vesicles needed to transmit a bit), respectively, on the basal *p_v_* for different levels of synaptic facilitation (different colors) at a canonical CA3 synapse ($N_{\text{max}}$ = 8). In general, energy use scales up with the basal probability of release ($p_{v}^{0}$) and with the strength of synaptic facilitation (*α_f_*), as expected (Fig. 4*B*, left). Notably, though, an increase in synaptic information transfer with stronger facilitation is accompanied by reduction in the synaptic energy efficiency, i.e., each released vesicle packs a smaller punch on average (Fig. 4*B*, right). The supralinear scaling of energy costs with synaptic information capacity implied by these examples suggests that in the context of realistic spiking patterns, individual CA3 synapses do not operate at optimal energy efficiency (according to the local measure of efficiency examined here), or minimize energy consumption; in fact, synapses lacking STP (Fig. 4*B*, black curves) require fewer releases per unit of information transmitted, albeit at significantly reduced overall information capacity, relative to dynamic synapses.

Do energy constraints, then, play no significant role in shaping the vesicle code at probabilistic hippocampal synapses? Examining the regime of stronger facilitation (*α_f_* $≳\alpha_{f}^{*}$) in the above example provides a potential clue in this regard. Figures 3*B*, 4*B* together indicate that a canonical synapse operating in the physiological range (*α_f_* $\approx \alpha_{f}^{*}$) transmits information at near-optimal capacity, and further increase in *α_f_* (by one order of magnitude, from 0.03 to 0.3 or 1) provides little additional benefit; the increased facilitation is, however, accompanied by a disproportionately larger increase in energy costs of synaptic transmission, which may be seen by comparing the green with the red/brown curves separately in Figures 3*B*, 4*B*. This specific example suggests that biological CA3 synapses may be poised to operate near the upper bound on information transfer rate while energy usage is economized to the extent that performance is not compromised.

To elaborate on the nature and generality of this energy-function trade-off, we compared biological STP synapses (*α_f_* ∼ $\alpha_{f}^{*}$) with synapses exhibiting weaker or stronger facilitation over approximately three decades of magnitude, estimating the relative change in the mean signaling capacity and mean energy cost per synapse when *α_f_* is scaled up or down by a factor of ∼10 relative to its physiological reference value ($\alpha_{f}^{*}$). Figure 4*C*, top, shows the distribution of relative changes over a range of model parameters (see Materials and Methods) for the specific example of $p_{v}^{0}$ = 0.03, and each cluster of data points represents a different comparison ($\alpha_{f}^{*}$ → *α_f_*). This aggregated data from our simulations indicates that stronger synaptic facilitation relative to the biological set-point provides little improvement in information transfer rates, but a relatively larger increase in energy expenditure; reducing facilitation, on the other hand, is associated with a sharp reduction in synaptic information capacity. The overall differences evident in Figure 4*C*, top, are found to be quite general and hold across a broad range of $p_{v}^{0}$ values examined (Fig. 4*C*, bottom).

The general trends suggested by Figure 4*C* are brought out clearly in Figure 4*D*, which shows how the normalized synaptic information capacity ($R_{\text{info}}$/$R_{\text{info}}^{*}$) and normalized average frequency of release events ($R_{ves}$/$R_{ves}^{*}$) vary with the strength of synaptic facilitation (*α_f_*). Each box (median ± IQR) summarizes the distribution of values for a particular $p_{v}^{0}$, and the different colored lines connect the median values corresponding to each choice of $p_{v}^{0}$. Simulations of our STP model suggest that synaptic information capacity is in general an increasing function of the strength of facilitation (*α_f_*), but saturates around the physiological level ($\alpha_{f}\approx \alpha_{f}^{*}$; Fig. 4*D*, top). Comparing it to Figure 4*D*, bottom, further increase in synaptic gain comes at a larger energy cost, bringing diminishing returns. By contrast, reducing facilitation below the biological operating point ($\alpha_{f}\approx \alpha_{f}^{*}$) by approximately one order of magnitude compromises synaptic channel capacity considerably, and the suppression of information transfer rates is particularly marked at smaller vesicular release probabilities ($p_{v}^{0}$). In sum, our results quantitatively demonstrate a novel form of local optimization embodied by STP of vesicular release at probabilistic CA3-CA1 synapses, and suggest that, under physiological conditions, individual synapses do not consume more resources than necessary while supporting highest-possible fidelity of information transmission over a wide range of presynaptic strengths.

## Discussion

Does an evolutionary drive toward energy-efficient signaling provide a relevant design principle to account for the salient properties of probabilistic transmitter release at individual hippocampal synapses? Previous investigations have focused on understanding energetic optimality at sensory pathway synapses (Laughlin et al., 1998; Harris et al., 2015, 2019; James et al., 2019). Given the diversity in synaptic morphology and tight structure-function relationships in synapses observed across brain areas, questions on synaptic design must be specific and addressed in a local context. In line with this, we examined STP at a cortical facilitating synapse, specifically the hippocampal Schaffer collateral-CA1 connection. Our synaptic model invoked detailed characterizing properties of single CA3 presynaptic terminals such as RRP size, release probability per vesicle and facilitation profiles derived from experiments and evaluated their impact on gating of realistic activity patterns. This allowed us to obtain biologically relevant insights into synapse-specific transmission properties in the hippocampus. Our results potentially suggest a normative account of biologically observed synaptic facilitation in terms of a local trade-off between two fundamental design constraints, vesicular information coding and energy utilization.

We estimated the capacity of a dynamic synapse, viewed as an unreliable channel, to relay behaviorally relevant temporal signals coded in presynaptic spiking activity via discrete vesicular release events. Our quantitative analysis shows how STF significantly improves the fidelity of synaptic information transduction. Remarkably, our simulations demonstrate that realistic STP enables vesicular release with widely different basal failure rates ($p_{v}^{0}$) to convey brief, variable high-frequency spike bursts with comparable fidelity; notably, this invariance is absent at static or weakly facilitating synapses, and is also not found in a phenomenological STP model used previously (Tsodyks and Markram, 1997; Pfister et al., 2010), highlighting the importance of incorporating synapse-specific experimental data to arrive at physiologically relevant findings about synaptic function. Further, physiological information rates over a broad range of release probabilities closely approach the predicted maximum capacity of a facilitating synapse of this type, i.e., within the limits imposed by the overall form of the model of presynaptic dynamics analyzed here. We estimated synaptic energy expenditure in terms of the average quantal release rate; this definition has been used previously to address energy efficiency of information processing in other neural contexts at both the synaptic and cellular levels (Levy and Baxter, 2002; Goldman, 2004; Harris et al., 2012; James et al., 2019). The metabolic cost of transmission at chemical synapses is primarily accounted for by pumps that reset the postsynaptic membrane potential and calcium transients to their resting values; additional but relatively smaller demands are made by reuptake of released glutamate from the synaptic cleft via the action of surrounding astrocytic glutamate transporters, and by the endocytic machinery involved in transmitter vesicle recycling and replacement at the presynaptic terminal (Harris et al., 2012). All of these energy-requiring processes scale up in direct proportion to the number of vesicular releases occurring, hence, the vesicle use, i.e., mean rate of release events, provides an accurate, equivalent measure of the net synaptic energy cost.

**Figure 5.**
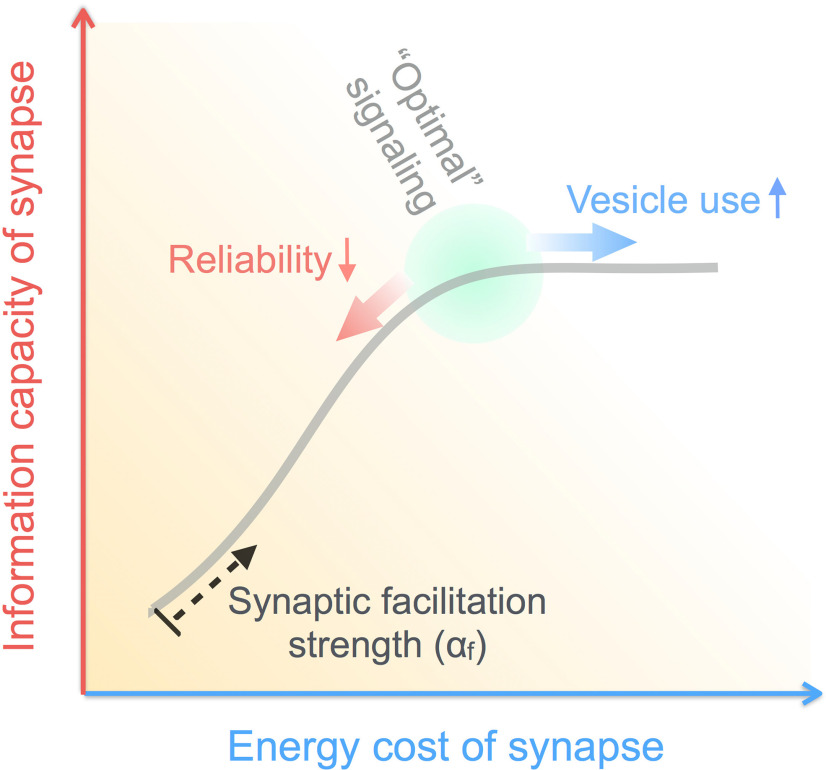
Activity-dependent STF regulates the cost-versus-capacity trade-off at unreliable CA3-CA1 synapses. The feasible “configurational space” of an STP synapse (schematically represented by the gray curve) is parametrized by the strength of synaptic gain, which constrains the relation between information transmitted across the synapse and the corresponding usage of synaptic resources. Our results suggest that biological synapses localize to the optimal set-point indicated in green.

Altogether, our results suggest a nuanced form of optimality that is at odds with maximization of synaptic efficiency (average number of quanta released per bit transmitted). Instead, our findings are consistent with the view that realistic STP synapses are poised, to within one order of magnitude in the gain parameter *α_f_*, to maintain near-maximal information transmission rates while penalizing excessive energy use (Fig. 5). Thus, we present evidence that energetic costs may also be important for regulating the properties of short-lived facilitation at low-release probability synapses. Interestingly, an analogous form of optimality was previously proposed in the context of the mammalian visual system (Vincent and Baddeley, 2003). Here, it was shown that synaptic energy restrictions can significantly shape early stimulus representations in the retina, and that the observed center-surround neural receptive fields provide the best balance between efficiency and performance, enabling near-maximal information transmission with largest possible synaptic energy savings. It remains to be seen, to what extent our findings are relevant to some of the other facilitating synapses in the mammalian central nervous system (Atluri and Regehr, 1996; Henze et al., 2002).

A key insight from our model is that synaptic information rates with physiological STP are nearly invariant to differences in the basal fusion probability per vesicle ($p_{v}^{0}$) that are present among individual CA3-CA1 synapses. This synaptic diversity, on the one hand, may represent the intrinsic, across-synapse differences in the ultrastructural details regulating transmitter release (Atwood and Karunanithi, 2002). On the other hand, a heterogeneous distribution of release probabilities may be a reflection of synaptically encoded memories, which are thought to be stored as distributed patterns of synaptic strength changes via activity-dependent long-term plasticity (McNaughton and Morris, 1987; Moser et al., 1998). Experimental evidence, besides theoretical considerations, suggests that both Hebbian and heterosynaptic plasticity in the hippocampus can have a presynaptic as well as postsynaptic locus of expression (Stevens and Wang, 1994; Enoki et al., 2009; Bliss and Collingridge, 2013; Letellier et al., 2016; Costa et al., 2017), which may, in part, be instantiated as persistent changes in $p_{v}^{0}$. Additionally, variation in presynaptic strengths may arise as a consequence of homeostatic (Branco et al., 2008; Zhao et al., 2011; Davis and Müller, 2015; Soares et al., 2017) or neuromodulatory (Qian and Saggau, 1997; Fernández de Sevilla and Buño, 2003) regulation of presynaptic calcium influx. In summary, several ongoing processes likely underlie the observed dispersion in CA3 presynaptic efficacies. Our analysis suggests that realistic STP dynamics operates at a set-point that compensates for this synaptic heterogeneity to support stable information rates. This implies a possible mechanism to insulate the dynamic synaptic interactions shaping short-timescale information processing of behaviorally evoked activity in the hippocampal circuit from the slower, longer-term adaptive changes that may be happening at these synapses because of learning or homeostatic adjustments. However, STP does not preclude other functional effects of enduring changes in synaptic efficacy, which can, e.g., continue to crucially affect retrieval of stored network patterns through associative recall, promote replay of activity sequences generated during navigational tasks that contributes to spatial memory consolidation, or govern the homeostatic regulation of neuronal activity levels. While the focus of our work is the presynaptic terminal, the long-term up or down-regulation of transmission at Schaffer collaterals can, in fact, occur postsynaptically as well, as shown by numerous experimental studies (Malenka and Bear, 2004); the postsynaptic changes, not explicitly considered here, would not directly impact our analysis of information capacity of presynaptic transmitter release. Overall, our findings add to the functional repertoire of short-term synaptic dynamics, which complements longer-lasting plasticity mechanisms to significantly enhance the functional complexity of biological synapses.

The use of synaptic resources versus the information transmitted is constrained by the synaptic gain parameter *α_f_*, which essentially controls the steepness of the facilitation profile for low release probabilities (Fig. 1*B*, left) and decides the operating point of the ensemble of CA3 synapses (Figs. 4*D*, 5). *α_f_* may be tuned over evolutionary timescales to some suitable optimum determined by the relative influence of different, competing selective pressures. This aligns with recent understanding of the evolutionary diversification of the synaptic proteome that may have contributed to functional specializations in brain areas and emergent behavior (Emes and Grant, 2012). In the context of the biophysical machinery governing transmitter release, what does the parameter *α_f_* correspond to? The basal probability of spike-evoked release is governed by the synchronous activation of the fast calcium sensor, Synaptotagmin-1 (Syt1; Geppert et al., 1994; Fernández-Chacón et al., 2001). On the other hand, recent findings have identified a separate calcium sensor, Syt7, carrying a high-affinity binding site for Ca^2+^ but with relatively slower kinetics (Bacaj et al., 2013), which was shown to be essential for progressive synaptic facilitation at CA3-CA1 terminals during persistent stimulation but not for the initial (basal) synaptic response (Jackman et al., 2016). Efficacy of its interaction with the protein machinery mediating vesicle fusion, or the kinetic parameters governing its sensitivity to calcium, could thus provide a possible biophysical basis to interpret the parameter *α_f_*. Alternately, kinetic parameters regulating calcium-induced calcium release from intracellular stores which has been implicated in enabling STF at hippocampal synapses (Emptage et al., 2001; Zhang et al., 2009), or developmental parameters regulating the relative arrangement of calcium channels and the release machinery (Nadkarni et al., 2012; Vyleta and Jonas, 2014), may determine the magnitude of *α_f_*. Biophysically detailed computational models of presynaptic calcium dynamics (Nadkarni et al., 2010; Hamid et al., 2019), outside the scope of the present study, can potentially shed more light on the molecular underpinnings of STP approximated by the reduced description in Equation 3 and help suggest mechanistic interpretations of *α_f_*.

Although our study specifically examines the role of local constraints in shaping synaptic release properties, it is expected that synapse design also carries imprints of selection pressures at higher levels of neural organization. A number of previous studies have elaborated on the functional implications of synaptic STP for collective dynamics on neuronal networks (Levina et al., 2007; Mongillo et al., 2008; Mejias and Torres, 2009). It is thus plausible that properties of individual synapses reflect such system-level design considerations as well. The present work, in particular, does not account for the typical RRP size of CA3 synapses, which is experimentally found to be close to ∼10 vesicles per bouton (Dobrunz and Stevens, 1997; Schikorski and Stevens, 1997). Our analysis, in fact, indicates that average synaptic information capacity is a monotonically increasing function of the size of the RRP; thus, if information transfer is to be improved, larger synapses ought be favored, which runs somewhat counter to the limit on synapse size reported by experiments. We surmise that an optimal RRP size might represent a compromise between reliability of signaling at individual synapses and information processing capacity on the network level. Given strong constraints on neural volume (or equivalently, on total availability of synaptic resources) as proposed previously (Laughlin and Sejnowski, 2003; Varshney et al., 2006), cortical connectivity might trade off high-fidelity synaptic transmission (proportional to the RRP size) for increased network complexity from a higher density of smaller, albeit less reliable, synapses (based on scaling arguments; Newman, 1988; Chklovskii et al., 2002). Detailed analysis of network information processing under physical constraints will be needed to evaluate the role of such an interaction across scales in shaping the design of fundamental processing elements in the brain.

To conclude, we propose that quantitative properties of probabilistic vesicular release at individual hippocampal synapses can be meaningfully interpreted in terms of a local cost-versus-capacity trade-off. The present analysis for a synapse in a higher brain circuit crucial to learning resonates with a growing body of work on the generality of information processing principles in understanding diverse cellular processes (Tkačik and Bialek, 2016). Our results suggest that the design of single synapses is primarily adapted to ensure optimal performance for diverse synaptic strengths, and this is achieved in an energetically cost-effective manner.

## Synthesis

Reviewing Editor: Alexey Semyanov, Shemyakin-Ovchinnikov Institute of Bioorganic Chemistry Decisions are customarily a result of the Reviewing Editor and the peer reviewers coming together and discussing their recommendations until a consensus is reached. When revisions are invited, a fact-based synthesis statement explaining their decision and outlining what is needed to prepare a revision will be listed below. The following reviewer(s) agreed to reveal their identity: Julia Harris.

Your manuscript has been seen by two reviewers and they agreed that it examines an exciting and essential topic. Using computational modeling grounded in physiological data, the authors address a question that has not yet been answered in the study of energy efficiency in synaptic communication: how is it possible for synaptic properties to be arranged such that they maximize information transmitted per energy used, but also remain flexible to allow for long-term changes in response to learning and experience? The present results suggest that short-term facilitation at the CA3-CA1 hippocampal synapse - a synapse famous for its long-term plasticity changes - allows for consistent information transfer across a range of presynaptic strengths, while the absence of short-term facilitation would entail an information rate that is highly dependent on release probability.

The manuscript provides two significant advances to the field of brain energetics and information processing. First, while examinations of the energy-information trade-off at synapses have previously focused on sensory pathway synapses, this paper examines a central, subcortical synapse, with high information processing power (CA3 to CA1). Secondly, previous studies of synaptic information transfer efficiency have typically left the puzzle of how to incorporate the phenomenon of synaptic plasticity unaddressed. In the present study, the authors have specifically investigated the effects of short-term plasticity on the energy efficiency of information transmission across synapses, providing insight into how synapses could consistently operate in an energy-efficient regime, despite long-term changes in their overall strength due to learning and experience.

The reviewers have made several specific comments which are put together below.

Major comments:

1. The pattern of presynaptic information is critical for this type of work. Please gives more details about the probability function of the presynaptic signal. The manuscript needs extra computations for other standards deviations of the input signal and auto-correlation functions, and not only for the delta correlation function as authors use in the computations. Such calculations with various input functions are necessary to expand the use of this theory for a larger number of input signals.

2. How does the input signal relate to the presynaptic neuron excitability, and how can neuron excitability change the presynaptic input pattern affecting the synaptic information transmission?

3. A limitation of this manuscript is a definition of energy as a number of release vesicles. The failure of release also requires a lot of energy in term of presynaptic calcium and other types of ions, and need to be considered in your calculations.

4. A single vesicle release is not enough to generate the postsynaptic action potential that is critical for transmitting information to another cell. How the graded potentials used in the manuscript relate to the postsynaptic information?

5. While this paper addresses a difficult question, a significant mystery is still unsolved. The question is (as mentioned above is): how is it possible for synaptic properties to be arranged such that they maximize information transmitted per energy used but also remain flexible to allow for long-term changes in response to learning and experience? The answer here is that, as long as they have some threshold level of short-term facilitation, it is possible for synapses to operate in a regime that maintains information transmission without ludicrous gains in energetic cost, regardless of the specific presynaptic release properties. Thus, information transmission is invariant to the heterogeneity of synaptic strengths that result from long-term plasticity. This is a very attractive model, but leaves open the question of what, then, is the point of long-term plasticity changes, if not to change information rates? What is the point of learning if learning cannot alter the rate at which information is transferred from one synapse the next?

6. While the present analysis has focused on the presynaptic side, the CA3-CA1 synapse is famous for postsynaptic elements of long-term plasticity. It would be informative to learn how the authors view their pre-synaptic results fitting into the plasticity of the synapse as a whole.

7. Critical parameters in this model relate to the passage of the animal through place fields: the input signal is considered to be bursts as the animal passes through a place field. Time is divided into steps, which are taken to be the fixed duration of every pass. It is unclear whether the length of this time-step was chosen based on cellular parameters (e.g., length of typical bursts/size of place fields) and/or behavioral parameters (e.g., the average running speed of mouse). Additionally, the analysis seems to assume that the animal is moving at a constant rate - what happens if the animal stays in the same place field for a length of time (and is there habituation in place cell firing?)

8. On Pg 6, we are told how Ps (synaptic release probability) and Pv (fusion probability per docked vesicle) are related - the non-linearity of which depends on the size of the RRP at the release site. Below this definition, we are given the assumption that “every spike can trigger the release of at most one vesicle per synapse.” So the size of the RRP is effectively 1? In this case, Ps would be linearly related to Pv, and therefore I would not understand the reason to focus on Pv (rather than Ps) for the majority of the figures.

9. The authors investigate a range of Pv across 4 orders of magnitude, but what is the physiological range? Anything above Pv=0.1 would not vary with STF gain. In Fig 4C, Pv=0.03 is singled out - is this in reference to a physiological finding?

Minor comments

1. Ps is release probability, so confusing that it is sometimes accompanied by the words “failure rate” (e.g., pg 9), which would suggest that a higher value means a higher rate of failure, rather than the other way around.

2. Pg 12 - “...enhancing the ability of the synapse to selectively transmit information-carrying spike discharges.” - Assuming that single spikes are not informative. How valid is this?

3. Pg 24 states “... if information transfer is to be improved, larger synapses ought to be favoured, which runs somewhat counter to the limit on synapse size reported by experiments” - so is this feasible? Please give an idea of the actual limit.

4. STF and STP seem to be used interchangeably, so preference for sticking to one.

5. Figures - use consistent color code (or dashed / not dashed) for STF vs no STF across figures

6. Fig. 1B. Typically PPR is a function of presynaptic calcium and is normally independent of the vesicle pool. The time scale of presynaptic calcium is around 50 - 100 ms. What is a biophysical mechanism modulating the release probability of the second vesicle after 400 ms?

7. “rescaled information rate” - explained on pg 13, but more informative axis label, e.g. information rate / max information rate across all Pv values.

8. Fig. 4B (right panel). If the point (Pg 18) is: “increase in synaptic information transfer with stronger facilitation is accompanied by a reduction in the synaptic energy efficiency...” Why not plot efficiency instead of 1/efficiency? This would also look less redundant compared to the left panel. How many trials have authors used to compute the statistic of synaptic dynamics with the release probability of 0.0001?
